# Prevalence of Vitamin D Deficiency in Elderly Patients Admitted to an Inpatient Rehabilitation Unit in Tropical Singapore

**DOI:** 10.1155/2016/9689760

**Published:** 2016-02-22

**Authors:** Jong J. Neo, Keng H. Kong

**Affiliations:** Department of Rehabilitation Medicine, Tan Tock Seng Hospital, Jalan Tan Tock Seng, Singapore 308433

## Abstract

*Background*. Data on hypovitaminosis D in elderly patients admitted to rehabilitation units in tropical countries are scarce.* Objective*. To assess the prevalence of vitamin D deficiency and its associated risk factors in elderly patients admitted to a rehabilitation unit in tropical Singapore.* Methods*. Prospective, cohort study of 134 subjects ≥ 65 years old admitted to a tertiary rehabilitation centre. Serum 25-hydroxyvitamin D3 (25OHD3) was measured on rehabilitation admission.* Results*. Mean age was 72.0 ± 5.7 years, and stroke was the commonest rehabilitation diagnosis (49.3%). Low 25OHD3 levels were present in 115 patients (85.6%) and 59 patients (44%) were deemed to be vitamin D deficient. The mean PTH level was significantly higher in patients with low 25OHD3 levels. (*p* = 0.002) Age, gender, vitamin D supplementation, premorbid ambulatory status, and admission/discharge Functional Independence Measure scores were not significantly associated with vitamin D deficiency. Malays/Indians (*p* = 0.013) and recurrent fallers (*p* = 0.029) were at significantly higher risk of vitamin D deficiency.* Conclusions*. Despite the tropical weather, vitamin D deficiency is common in elderly subjects admitted to a rehabilitation unit in Singapore. Routine assessment of vitamin D levels is recommended especially in those with a history of recurrent falls and patients of Indian/Malay ethnicity.

## 1. Introduction

Vitamin D is essential for overall health and well-being. It is produced by our skin upon exposure to ultraviolet B irradiation from sunlight. More than 90% of circulating vitamin D originates from cutaneous production. Its role in various medical conditions such as osteoporosis, cancer, diabetes mellitus, and infection has been well studied [1]. Vitamin D deficiency is also associated with sarcopenia, muscle weakness, a reduced ability to perform activities of daily living, increased body sway, and increased risk of falls and hip fractures [1–4].

Patients who require inpatient rehabilitation stay after an acute hospitalisation possess multiple risk factors for vitamin D deficiency. Frequently, these patients are older and medically complex and suffer from reduced mobility and poor nutritional status. The elderly are particularly at risk of vitamin D deficiency due to factors like decreased dermal production of vitamin D following exposure to sunlight because of atrophic skin changes [5], lower dietary vitamin D content, impaired gastrointestinal absorption, and decreased renal production of 1,25OH_2_D [1].

Given this, there is a case to be made for the screening of vitamin D levels in rehabilitation inpatients, so that vitamin D supplementation can be given to patients with low vitamin D levels. Recent studies of patients admitted to rehabilitation units showed that hypovitaminosis D is common with rates ranging from 68.9% to 94% [6–9]. In a study of 315 patients with fractured neck of femur admitted to a rehabilitation hospital, Di Monaco et al. found that 75% had frank vitamin D deficiency (25OHD level less than 12 ng/mL) [6]. Shinchuk et al. evaluated 53 patients with varying diagnoses admitted to a subacute rehabilitation facility and reported vitamin D insufficiency (25OHD level less than 30 ng/mL) and vitamin D deficiency (25OHD level less than 20 ng/mL) in 83% and 49.1% of patients, respectively [7]. Kiebzak et al. studied 100 acute inpatient rehabilitation patients with a diagnosis of fracture, hip or knee replacement, and stroke and found 11% with vitamin D deficiency (25OHD level less than 8 ng/mL) and 94% with insufficiency (25OHD level less than 32 ng/mL) [8]. Pellicane et al. in a retrospective review of 101 patients reported that 68.9% were insufficient (25OHD level less than 30 ng/mL) and 8.1% were deficient (25OHD less than 10 ng/mL) [9]. In 2 of these studies, vitamin D level was significantly correlated to functional status on admission to and discharge from rehabilitation [6, 8].

However, these studies were conducted in temperate countries, involving predominantly Caucasian patients, in whom it has been previously shown that vitamin D insufficiency is more prevalent due to reduced cutaneous synthesis during winter months [10]. Studies examining the prevalence of vitamin D deficiency in rehabilitation inpatients in tropical countries are scarce. Singapore is a tropical, multiracial country consisting predominantly of 74.1% Chinese, 13.4% Malays, and 9.2% Indians with sunny weather throughout the year [11].

The objectives of this study were to investigate the prevalence of vitamin D deficiency in a cohort of elderly patients admitted to a rehabilitation centre in Singapore and to establish demographic and clinical factors associated with vitamin D deficiency.

## 2. Methods

### 2.1. Study Design, Setting, and Population

This was a prospective study of hospitalized patients ≥65 years admitted to a 90-bedded tertiary rehabilitation facility over a 12-month period from January to December 2012. The rehabilitation centre has 4 major programmes: stroke rehabilitation, traumatic brain injury rehabilitation, spinal injury rehabilitation, and musculoskeletal rehabilitation (including polytrauma, lower extremity joint replacements, and lower limb amputation). Excluded were patients with known primary hyperparathyroidism and patients on dialysis as dialysis can affect serum vitamin D levels.

### 2.2. Data Collected

Data collected included patient demographics (age, gender, ethnicity, and body mass index), premorbid ambulatory status using the Functional Ambulation Category (FAC) [12], frequency of falls over the past 2 years prior to current hospitalisation, diagnosis on rehabilitation admission, medical comorbidities, vitamin D supplementation before hospital admission (defined as taking supplementation for at least 5 days a week for the past one month as reported by the patient), rehabilitation length of stay, and functional status on admission to and discharge from rehabilitation as measured on the Functional Independence Measure (FIM) [13]. The FAC scores from 0 to 5, with 0 indicating inability to walk and 5 indicating independent ambulation. Fall history was categorized as “no falls/once-only fallers” and “recurrent fallers,” as evidence suggests that once-only fallers are characteristically more closely related to nonfallers than recurrent fallers. Reaction time, quadriceps strength, and postural sway are worse in recurrent fallers than nonfallers and once-only fallers [14]. FIM efficiency was calculated by dividing the admission and discharge FIM scores by the rehabilitation length of stay.

Nonfasting venous blood was withdrawn for 25-hydroxyvitamin D3 (25OHD3) level within 7 days of rehabilitation admission. It was measured using Roche Elecsys vitamin D3 assay (Roche Mannheim, Germany) in the hospital's accredited laboratory. Serum calcium, phosphate, albumin, and parathyroid hormone (PTH) levels were also measured. At present, there is no consensus on the definition of vitamin D deficiency. The Institute of Medicine has defined serum vitamin D levels of 20 mg/L as adequate and less than 12 mg/L as deficient [15]. On the other hand, Holick and the Endocrine Society Clinical Practice Guideline defined vitamin D deficiency as <20 mg/L and insufficiency as 21 to 29 mg/L [1, 16]. The arguments on the scientific merits of these definitions have been presented elsewhere [17, 18]. In this study, we defined vitamin D deficiency/insufficiency using Holick classification as follows: vitamin D deficiency is defined as 25OHD3 level <20 ng/mL and vitamin D insufficiency as 25OHD3 level of 21 to 29 ng/mL [1].

Informed consent was obtained from all patients and the study was approved by the institution's ethics board.

### 2.3. Statistical Analysis

We analyzed the data using SPSS version 16. We present continuous variables as mean and standard deviation, while categorical data are presented as frequency and percentages. Univariate logistic regression analysis was used to ascertain factors associated with vitamin D deficiency. The factors studied were age, gender, ethnicity, body mass index, vitamin D supplementation, premorbid ambulatory as measured on the FAC, recurrent fallers (history of ≥2 falls in the previous 2 years before admission), and admission and discharge FIM scores and FIM efficiency. Significant factors from univariate logistic regression were then entered into a multivariate logistic regression analysis to ascertain independent factors associated with vitamin D deficiency. Odds ratio and 95% confidence intervals were also calculated. We considered a *p* value of <0.05 (2-sided) to be statistically significant.

## 3. Results

A total of 276 patients were screened during the study period. Thirty-six patients were excluded as they were medically unwell, for example, fever and deep vein thrombosis, and 53 were unable to give consent because of language and/or cognitive deficits. Consent was sought from a total of 240 eligible patients of whom 134 agreed to participate (refusal rate of 44.1%).

Patient characteristics are shown in Table 1. The mean age was 72.0 ± 5.7 years and there was a slight preponderance of male patients (58.2%). Ethnically, the majority of patients were Chinese (88.1%). Stroke was the commonest rehabilitation diagnosis (49.3%) followed by polytrauma (17.9%), spinal cord injury (15.7%), and hip/knee joint replacement (14.9%). Premorbidly, the majority of patients (93.3%) were community ambulators with a median FAC score of 5. The patients stayed on an average of 11.8 ± 9.6 days in the acute facility before admission to rehabilitation. Almost one-third (26.9%) of patients were on vitamin D supplements. No patient was on antiepileptics. Slightly more than one-third (35.8%) of patients had a history of at least one fall over the previous 2 years prior to admission and 18.7% were deemed to be recurrent fallers (defined as ≥2 falls). The mean FIM scores on admission to and discharge from rehabilitation were 78.24 ± 18.5 and 92.3 ± 18.8, respectively, and the mean FIM efficiency score was 0.59 ± 0.56.

The 25OHD3 levels in the study cohort ranged from 4.0 ng/mL to 42.0 ng/mL, with a mean of 21.4 ± 7.8 ng/mL. One hundred and fifteen (88.5%) patients had hypovitaminosis D of whom 59 (44.0%) patients were vitamin D deficient and 56 (41.8%) patients were vitamin D insufficient. There was no significant difference in mean 25OHD3 levels between the different diagnosis groups (*t*-test, *p* = 0.32).  Table 2 shows the mean PTH and adjusted calcium levels in vitamin D deficient, vitamin D insufficient, and normal patients. There was no statistical difference in adjusted mean calcium levels across all 3 groups of patients. However, mean PTH level was significantly higher in vitamin D deficient and insufficient patients.

Univariate analysis of factors related to vitamin D deficiency is shown in Table 3. Age, gender, vitamin D supplementation, premorbid ambulatory status, and scores on admission FIM, discharge FIM, and FIM efficiency were not significantly associated with vitamin D deficiency. Only race and recurrent fallers were significantly associated with vitamin D deficiency. Malay and Indian patients were more likely to be at risk of vitamin D deficiency than Chinese patients. Among recurrent fallers, those with ≥3 falls had significantly lower mean 25OHD3 levels than patients with 2 falls (15.9 ± 7.4 ng/mL compared with 20.8 ± 1.7 ng/mL; *p* = 0.006). On multivariate logistic analysis, race and recurrent fallers remained significant independent factors associated with vitamin D deficiency (see Table 4).

## 4. Discussion

In this study of 134 elderly inpatients with various diagnoses admitted to a rehabilitation centre in Singapore, 85.5% of patients had hypovitaminosis D, of whom 44.0% were vitamin D deficient (25OHD3 < 20 ng/mL) and 41.8% were vitamin D insufficient (25OHD3 21 to 29 ng/mL). This high prevalence of hypovitaminosis D is comparable to studies of patients admitted to inpatient rehabilitation services in the temperate countries. Using almost similar 25OHD levels to define hypovitaminosis D, Shinchuk et al. reported prevalence rates ranging from 68.4% to 94% [7–9]. The 44.0% prevalence rate of vitamin D deficiency in our study is similar to the 49.1% reported by Shinchuk et al. but much higher than the 11.0% and 8.1% reported by Kiebzak et al. and Pellicane et al., respectively. The reason for the difference lies in the definition of vitamin D deficiency used by Kiebzak et al. and Pellicane et al. They had defined vitamin D deficiency as 25OHD level <8 ng/mL and 10 ng/mL, respectively. If vitamin D deficiency was defined as <10 ng/mL in our study, its prevalence would be 7.5% instead.

The prevalence of vitamin D deficiency and insufficiency is not dissimilar from another local study examining hypovitaminosis in the elderly. In a cross-sectional study of 218 elderly patients (mean age 86.6 years) admitted to a geriatrics acute unit by Goh et al. [19], the prevalence of vitamin D deficiency and insufficiency was 40.8% and 27.7%, respectively. Hypovitaminosis D is also prevalent in the Middle East and other tropical countries like Malaysia, Indonesia, and Vietnam [20–22].

Of the factors studied for association with vitamin D deficiency, only race and recurrent fallers were found to be significant. Vitamin D deficiency was significantly more common in Malay and Indian patients than Chinese patients. 71.4% and 77.7% of Malay and Indian patients had Vitamin D deficiency compared to 39.8% of Chinese patients. This racial discrepancy in vitamin D status was also reported in a previous study involving healthy adult Singaporeans [23]. It is likely that the darker pigmentation of Malay/Indian patients contributed to this finding. Similar observations have also been reported in other multiracial populations. In a study by Forrest and Stuhldreher involving adults aged 20 years and above in the United States, suboptimal vitamin D level was most prevalent in African Americans, followed by Hispanics and non-Hispanic whites [24]. Subjects with darker skin pigmentation have been shown to have a lower 25OHD level after ultraviolet B radiation exposure, because melanin in the skin absorbed and competed with 7-dehydrocholesterol for ultraviolet B photons [25, 26]. Genetic factors may also play a role and some Indians have been demonstrated to have increased renal 24,25(OH)_2_D-hydroxylase activity which facilitates the production of inactive 24,25(OH)_2_D at the expense of 1,25(OH)_2_D [27].

The association between falls and vitamin D deficiency has been reported previously [1]. This association in our study is further strengthened by the finding that patients with ≥3 falls had significantly lower 25OHD3 levels than those with 2 falls. Vitamin D deficiency causes atrophy of type II muscle fibres which are recruited first to prevent a fall and proximal myopathy [28]. The effectiveness of vitamin D in preventing falls in the elderly is supported by a recent meta-analysis [29].

Although advanced age and obesity (high BMI) have been previously reported to be associated with vitamin D deficiency [1] we were not able to demonstrate a correlation between high BMI and vitamin D deficiency, likely due to the fact that the mean BMI in our study cohort was 23.2 ± 3.7, indicating that most patients were not obese.

The relationship between vitamin D levels and admission/discharge FIM scores and FIM efficiency was explored in 2 previous studies. In the first study, Shinchuk et al. [7] found weak but statistically significant associations between serum vitamin D level and admission FIM (*r* = 0.25, *p* = 0.011) and discharge FIM scores (*r* = 0.23, *p* = 0.021). There was also a significant difference in FIM efficiency scores comparing patients with vitamin D levels higher and lower than the median 25OHD level (16.6 ng/mL; 2.0 ± 1.1 versus 1.6 ± 0.9; *p* = 0.026). In the second study, Pellicane et al. [9] found statistically significant difference in unadjusted FIM efficiency scores between vitamin D sufficient and vitamin D insufficient/deficient patients. However, when FIM efficiency scores were adjusted for demographic and clinical factors, patient diagnosis, not vitamin D level, was a more important predictor of FIM efficiency. In our study, no statistical difference in admission/discharge FIM and FIM efficiency scores was seen between vitamin D deficient and non-vitamin D deficient patients. It is possible that differences in case-mix could have contributed to this finding.

There was no significant difference in vitamin D levels between those who reported vitamin D supplementation and those who did not. Possible reasons include compliance with supplementation, and/or inadequate supplementation, either because the duration of supplementation was too short or because the strength of supplements taken was insufficient. It is also possible that if vitamin D supplements taken were vitamin D2 (ergocalciferol), these would not have been detected as we only assayed 25OHD3.

The inverse relationship between vitamin D and PTH levels observed in our study is well established [30]. Vitamin D and PTH are both responsible for maintaining extracellular calcium homeostasis. Vitamin D increases the efficiency of intestinal calcium absorption, and PTH is secreted in response to low-circulating calcium concentrations or vitamin D. Elevated PTH secondary to low vitamin D increases calcium resorption from the skeleton at the expense of an increased risk of fracture [31].

### 4.1. Study Limitations

There are several study limitations. Firstly, the sample size was relatively small and the refusal rate for participation in this study was rather high, at 44.1%. However, demographic characteristics and rehabilitation diagnoses in these patients were not significantly different from those who participated in the study. Secondly, dietary vitamin D intake and sunlight exposure were not captured. Finally, 25OHD3 levels were measured rather than total 25OHD, as that was the only vitamin D assay available at our hospital when the study was conducted. However, this is not likely to have a significant impact on our study results as more than 95% of 25OHD measurable in the serum is 25OHD3 [32].

## 5. Conclusion

Despite the presence of sunshine all year round in Singapore, the prevalence of hypovitaminosis D is not any lower in this cohort of elderly rehabilitation inpatients compared to studies conducted in temperate countries. Given the impact of vitamin D on reducing falls, improving muscle strength and function, routine laboratory assay of vitamin D level is recommended in this group of patients, especially in those with a history of recurrent falls and patients of Indian/Malay ethnicity.

## Figures and Tables

**Table 1:** Baseline characteristics of patients.

Age, years (mean ± SD)	72.0 ± 5.69
Gender	
Male	78 (58.2%)
Female	56 (41.8%)
Race	
Chinese	118 (88.1%)
Indian	9 (6.7%)
Malay	7 (5.2%)
Diagnosis group	
Stroke	66 (49.3%)
Musculoskeletal	
Polytrauma	24 (17.9%)
Hip/knee joint replacement	20 (14.9%)
Spinal cord injury	21 (15.7%)
Traumatic brain injury	8 (6%)
Others	9 (6.7%)
Body mass index (BMI) (mean ± SD)	23.2 ± 3.7
Premorbid FAC score (median ± IQR)	5 (0)
Premorbid ambulation status	
Independent (community)	125 (93.3%)
Independent (home-bound)	3 (2.2%)
Assisted	6 (4.5%)
Common medical comorbidities	
Hypertension	103 (76.9%)
Diabetes mellitus	53 (39.6%)
Hyperlipidaemia	100 (71.6%)
Osteoarthritis	46 (34.3%)
Calcium and/or vitamin D supplementation	36 (26.9%)
History of fall in the last 2 years	48 (35.8%)
Recurrent fallers (≥2 falls in the last 2 years)	25 (18.7%)
Length of stay in acute facility, days (mean ± SD)	11.8 ± 9.6
Length of stay in rehabilitation, days (mean ± SD)	26.8 ± 8.0
Admission FIM score(mean ± SD)	78.2 ± 18.5
Admission FIM motor score	47.6 ± 14.1
Admission FIM cognition score	30.7 ± 6.7
Discharge FIM score (mean ± SD)	92.3 ± 18.8
Discharge FIM motor score	60.6 ± 15.0
Discharge FIM cognition score	31.4 ± 5.8
FIM efficiency score (mean ± SD)	0.59 ± 0.56
25OHD3 level, ng/mL (mean ± SD)	21.4 ± 7.8
Vitamin D deficiency (<20 ng/mL)	59 (44.0%)
Vitamin D insufficiency (21 to 29 ng/mL)	56 (41.8%)
Normal vitamin D level (≥30 ng/mL)	19 (14.2%)
Adjusted calcium level, mmol/L (mean ± SD)	2.41 ± 0.09
Parathyroid hormone level, pmol/L (mean ± SD)	4.08 ± 2.10

FAC: Functional Ambulation Category,

FIM: Functional Independence Measure,

SD: standard deviation,

IQR: interquartile range.

**Table 2:** Mean ± standard deviation of parathyroid hormone (PTH) and adjusted calcium levels.

	Vitamin D deficiency	Vitamin D insufficiency	Normal	*p* value
PTH (pmol/L)	4.19 ± 2.16	4.22 ± 2.15	2.97 ± 1.03	0.002
Calcium (mmol/L)	2.44 ± 0.87	2.41 ± 1.06	2.39 ± 0.77	0.14

**Table 3:** Univariate analysis of factors associated with vitamin D deficiency.

Factor	Odds ratio (95% confidence interval)	*p* value
Age	1.03 (0.96–1.10)	0.31
Gender (female)	0.61 (0.30–1.22)	0.16
Race (Malays/Indians versus Chinese)	0.22 (0.07–0.73)	0.013
Body mass index	0.98 (0.82–1.09)	0.71
Recurrent fallers (yes versus no)	0.36 (0.14–0.90)	0.029
FAC score	1.54 (0.80–2.91)	0.19
Premorbid vitamin D supplementation (yes versus no)	0.99 (0.46–2.15)	0.99
Admission FIM score	−3.27 (−9.60–3.00)	0.31
Discharge FIM score	−3.69 (−1015–2.77)	0.26
FIM efficiency	−0.34 (−0.18–1.220)	0.69

**Table 4:** Multivariate analysis of factors associated with vitamin D deficiency.

Factor	Odds ratio (95% confidence interval)	*p* value
Race (Malays/Indians versus Chinese)	0.21 (0.06–0.70)	0.011
Recurrent fallers (yes versus no)	2.87 (1.13–7.21)	0.025
